# GS-E3D, a new pectin lyase-modified red ginseng extract, inhibited diabetes-related renal dysfunction in streptozotocin-induced diabetic rats

**DOI:** 10.1186/s12906-017-1925-7

**Published:** 2017-08-29

**Authors:** Chan-Sik Kim, Kyuhyung Jo, Jin Sook Kim, Mi-Kyung Pyo, Junghyun Kim

**Affiliations:** 1 Korean Medicine Convergence Research Division, Korea Institute of Oriental Medicine (KIOM), 1672 Yuseongdaero, Yuseong-gu, Daejeon, 34054 South Korea; 2 International Ginseng and Herb Research Institute, 25 Insamgwangjang-ro, Geumsan-eup, Geumsan-gun, 32724 South Korea; 3 Department of Oral Pathology, School of Dentistry, Chonbuk National University, 567 Baekjedaero, Jeonju, 54896 South Korea

**Keywords:** Advanced glycation end-products, Diabetic nephropathy, GS-E3D, Red ginseng

## Abstract

**Background:**

GS-E3D is a newly developed pectin lyase-modified red ginseng extract. The purpose of this study was to investigate the therapeutic effects of GS-E3D on diabetes-related renal dysfunction in streptozotocin-induced diabetic rats.

**Method:**

GS-E3D (25, 50, and 100 mg/kg body weight per day) was administered for 6 weeks. The levels of blood glucose and hemoglobin A1c, and of urinary albumin, 8-hydroxy-2′-deoxyguanosine (8-OHdG), and advanced glycation end-products (AGEs) were determined. Kidney histopathology, renal accumulation of AGEs, and expression of α-smooth muscle actin (α-SMA) were also examined.

**Results:**

Administration of GS-E3D for 6 weeks reduced urinary levels of albumin, 8-OHdG, and AGEs in diabetic rats. Mesangial expansion, renal accumulation of AGEs, and enhanced α-SMA expression were significantly inhibited by GS-E3D treatment. Oral administration of GS-E3D dose-dependently improved all symptoms of diabetic nephropathy by inhibiting renal accumulation of AGEs and oxidative stress.

**Conclusion:**

The results of this study indicate that the use of GS-E3D as a food supplement may provide effective treatment of diabetes-induced renal dysfunction.

## Background

Diabetic nephropathy is one of the most significant chronic complications of diabetes [1]. Hyperglycemia may enhance oxidative stress and inflammation in the renal tissues and lead to the development of kidney failure [2]. The current treatment strategy for patients with diabetic nephropathy is to prevent or delay disease progression by strict control of blood glucose [3]. Although various glucose-lowering drugs are clinically available, the prevalence of diabetic nephropathy has increased worldwide [4].

Reactive oxygen species (ROS) and advanced glycation end-products (AGEs) play major roles in the development and progression of diabetic kidney disease [5–9]. AGEs are heterogeneous sugar-derived irreversible protein modifications have been implicated in the pathogenesis of diabetes and other age-related diseases [6]. The formation of AGEs is accelerated under hyperglycemic conditions, as well as by oxidative stress. Oxidative stress, which is fueled by excessive ROS production during glucose autoxidation, drives the nonenzymatic, covalent attachment of glucose molecules to circulating proteins, thus forming AGEs [10]. Moreover, the receptor for AGEs (RAGE) has also been implicated in mediating AGE-induced renal damage [11]. The interaction between AGEs and RAGE can trigger ROS production and signaling pathways, leading to cell injury [12]. Oxidative stress arising from the interaction between AGEs and RAGE leads to apoptosis of renal glomerular cells [13] and podocytes [14].

AGE inhibitors such as aminoguanidine and LR-90 attenuate mesangial expansion and proteinuria in animal models of diabetes [15–17]. However, aminoguanidine has not been used clinically to treat diabetic nephropathy because of its adverse effects, which include pro-oxidant activities [18] and inhibition of nitric oxide synthase [19]. Some natural and synthetic compounds have been proposed to act as AGE inhibitors [20]. Generally, botanical products are often perceived to be safer than synthetic compounds. Therefore, interest in the use of herbal products has been increasing.

*Panax ginseng* is one of the most popular health foods and it has been used to increase vitality for centuries in Asian countries. Red ginseng, a product derived from *P. ginseng*, is manufactured by repetitive steaming and drying cycles [21]. This manufacturing process promotes the formation of additional beneficial compounds, known as ginsenosides. Red ginseng has shown potent pharmacological effects on the immune response, and on metabolic disease and cancer [22–26]. In addition, red ginseng has its long history of ethnopharmacological evidence for anti-diabetic use. Several investigations have revealed that red ginseng exerted anti-diabetic function in diabetic animals [27, 28] and diabetic patients [29]. Hong et al. showed that red ginseng lowered blood glucose levels and protected pancreatic ß-cell degeneration in streptozotocin (STZ)-induced diabetic mice [30]. Recently, our group showed that red ginseng decreased oxidative stress, AGEs accumulation and renal injury in D-galactose-induced aging rats [31]. More recently, Quan et al. showed that oral administration of red ginseng had a beneficial effect on AGEs-mediated renal injury in STZ-induced diabetic rats [32]. These previous in vitro and in vivo data suggest that red ginseng may exerts a number of beneficial activities, including anti-glycation, anti-oxidative and renoprotective effects under diabetic conditions.

Recently, some transformation methods have been applied to red ginseng, including enzymatic conversion [33] and fermentation [34]. These biotransformation processes increased the pharmacological potency of red ginseng in several animal models of disease [35–38]. Wang et al. reported that ginsenosides were metabolized by microorganisms in a biotransformation product of red ginseng, which improved their intestinal absorption, increased bioactivity, and diminished the toxicity of the metabolite [39]. Indeed, Kim et al. reported that a fermented red ginseng extract by *Lactobacillus fermentum* had a more potent anti-oxidative activities than normal red ginseng in vitro and increased anti-oxidant enzyme activities in STZ-induced diabetic rats [40]. GS-E3D is a newly developed pectin lyase-modified red ginseng extract. This product showed anti-obesity activity in a mouse model [41] and had anti-inflammatory effects on macrophage cells in vitro [42]. To the best of our knowledge, no reports have described the therapeutic effects of GS-E3D on diabetes-related renal dysfunction. To address this, we studied the effect of GS-E3D on diabetes-induced renal dysfunction in a streptozotocin-induced diabetic rat model. In addition, we tested the hypothesis that GS-E3D would provide effective inhibition of renal AGE accumulation in this animal model.

## Methods

### GS-E3D preparation

Four-year-old dried *P. ginseng* was purchased from a local market (Wooshin Industrial Co., Ltd., Geumsan, Korea). The botanical identification was made by botanist Dr. M. K. Pyo (International Ginseng and Herb Research Institute, Korea). Voucher specimen (No. GS201104) is deposited in the herbarium of the International Ginseng and Herb Research Institute (Kumsan, Korea). GS-E3D was prepared according to our previous report [41]. Briefly, red ginseng extract, adjusted to 6 Brix, was incubated with 10% pectin lyase (EC 4.2.2.10; Novozyme, #33095, Denmark) at 50 °C for 5 d in a shaking incubator (150 rpm). To terminate the reaction, the processed extract was incubated at 95 °C for 10 min and then freeze-dried for storage prior to the subsequent experiments. The dried GS-E3D consisted of 120.2 mg/g crude saponin, which contained the following ginsenosides: 5.9 mg/g Rg1, 12.6 mg/g Re, 4.7 mg/g Rf, 30.2 mg/g Rb1, 14.0 mg/g Rc, 17.6 mg/gRb2, 2.5 mg/g Rb3, 27.7 mg/g Rd., 1.3 mg/g 20(S)-Rg3, 1.4 mg/g 20(R)-Rg3, 0.8 mg/g Rk1, and 1.5 mg/g Rg5.

### Animals and experimental design

Seven-week-old male Sprague-Dawley with body weights of 200 ~ 225 g were purchased from Orient Bio (Seongnam, Korea) and acclimated for 1 week prior to the induction of diabetes by a single intraperitoneal injection of 60 mg/kg streptozotocin (STZ). Age-matched control rats received an equal volume of vehicle (0.01 M citrate buffer, pH 4.5). Rats were individually housed in plastic cages and maintained at 24 °C ± 2 °C with a 12 h light:dark cycle and received a standard pellet diet (Ralston Purina, St. Louis, MO, USA) and tap water ad libitum. One week after these injections, blood samples were obtained from the tail vein. Rats with a blood glucose level greater than 300 mg/dL were considered to be diabetic. The rats were then divided into 5 groups of 10 rats, as follows: (1) normal control rats (NOR); (2) STZ-induced diabetic rats (diabetes mellitus; DM); and (3, 4, and 5) STZ-induced diabetic rats treated with GS-E3D (25, 50, or 100 mg/kg body weight, respectively). GS-E3D was dissolved in distilled water and orally administered for 6 weeks, and the other groups were given the same amount of vehicle gavage for 6 weeks.

All experimental procedures were approved by the Institutional Animal Care and Use Committee of the Korea Institute of Oriental Medicine (Approval No. 15–100, Daejeon, Korea)

### Blood glucose and hemoglobin A1c (HbA1c) levels

Blood samples were collected from the tail vein after a 16-h fast. Blood glucose and HbA1c levels were measured using an automated analyzer (Wako, Tokyo, Japan).

### Quantification of urinary albumin, 8-Hydroxy-2′-Deoxyguanosine (8-OHdG), and advanced Glycation end-products (AGEs)

Individual rats were placed in metabolic cages for 24-h urine collection. Daily urinary albumin, 8-OHdG, and AGE levels were measured using a rat albumin enzyme-linked immunosorbent assay (ELISA) kit (Abcam, Cambridge, UK), an 8-OHdG Check ELISA kit (Cosmo Bio, Tokyo, Japan), and a rat AGEs ELISA kit (Cusabio, Wuhan, China), respectively.

### Histopathology

Renal tissues were fixed in 10% neutralized formaldehyde and embedded in paraffin prior to preparing 4-μm sections. The sections were stained with periodic acid-Schiff reagent (Sigma, St. Louis, MO, USA) and counterstained with hematoxylin. A total of 20 glomeruli were randomly selected from each rat and the glomerular tuft and mesangial matrix areas were measured using Image J software (National Institutes of Health, Bethesda, MD, USA).

### Immunohistochemical staining

Deparaffinized sections were hydrated and treated with 1% H_2_O_2_ in methanol prior to incubation with antibodies raised against either AGEs (1:200; Transgenic Inc., Kobe, Japan) or α-smooth muscle actin (α-SMA) (1:250; Santa Cruz Biotechnology, Paso Robles, CA, USA) for 1 h at room temperature. Signal detection was achieved using the Envision kit (DAKO, Carpinteria, CA, USA) and visualized by 3,3′-diaminobenzidine tetrahydrochloride. As a negative control, tissue sections were incubated with serum from non-immunized animals, instead of the primary antibody. The immunohistochemical signal intensity was analyzed in 20 randomly selected glomeruli from each rat using ImageJ software (National Institutes of Health, Bethesda, MD, USA).

### Statistical analysis

Group data were analyzed by one-way analysis of variance followed by Tukey’s multiple comparison test or by unpaired Student’s t-test, using GraphPad Prism 6.0 software (GraphPad, San Diego, CA, USA). Differences with a *p* value of <0.05 were considered statistically significant.

## Results

### Blood glucose control

Blood glucose levels are summarized in Table 1. The blood glucose and HbA1c levels were significantly increased in diabetic rats (*p* < 0.05). No differences in blood glucose or HbA1c levels were noted between GS-E3D-treated and vehicle-treated diabetic rats.

**Table 1:** Blood glucose and HbA1c levels

		NOR	DM	GS-E3D (mg/kg)		
				25	50	100
Blood glucose (mg/dL)	Initial	63.4 ± 6.1	307.6 ± 32.8*	307.5 ± 43.4	307.8 ± 45.7	301.6 ± 40.1
	Final	68.3 ± 14.8	335.3 ± 93.0*	327.8 ± 146.1	330.8 ± 75.8	349.8 ± 43.4
HbA1c (%)		4.17 ± 0.22	6.55 ± 0.37*	6.49 ± 0.28	6.62 ± 0.27	6.60 ± 0.35

*NOR* normal rat, *DM* STZ-induced diabetic rat, GS-E3D, DM treated with GS-E3D (25, 50, or 100 mg/kg). All data were expressed as mean ± standard deviation (*n* = 10); *p < 0.05 vs. NOR group

### Renal histopathology and Albuminuria

**Fig. 1:**
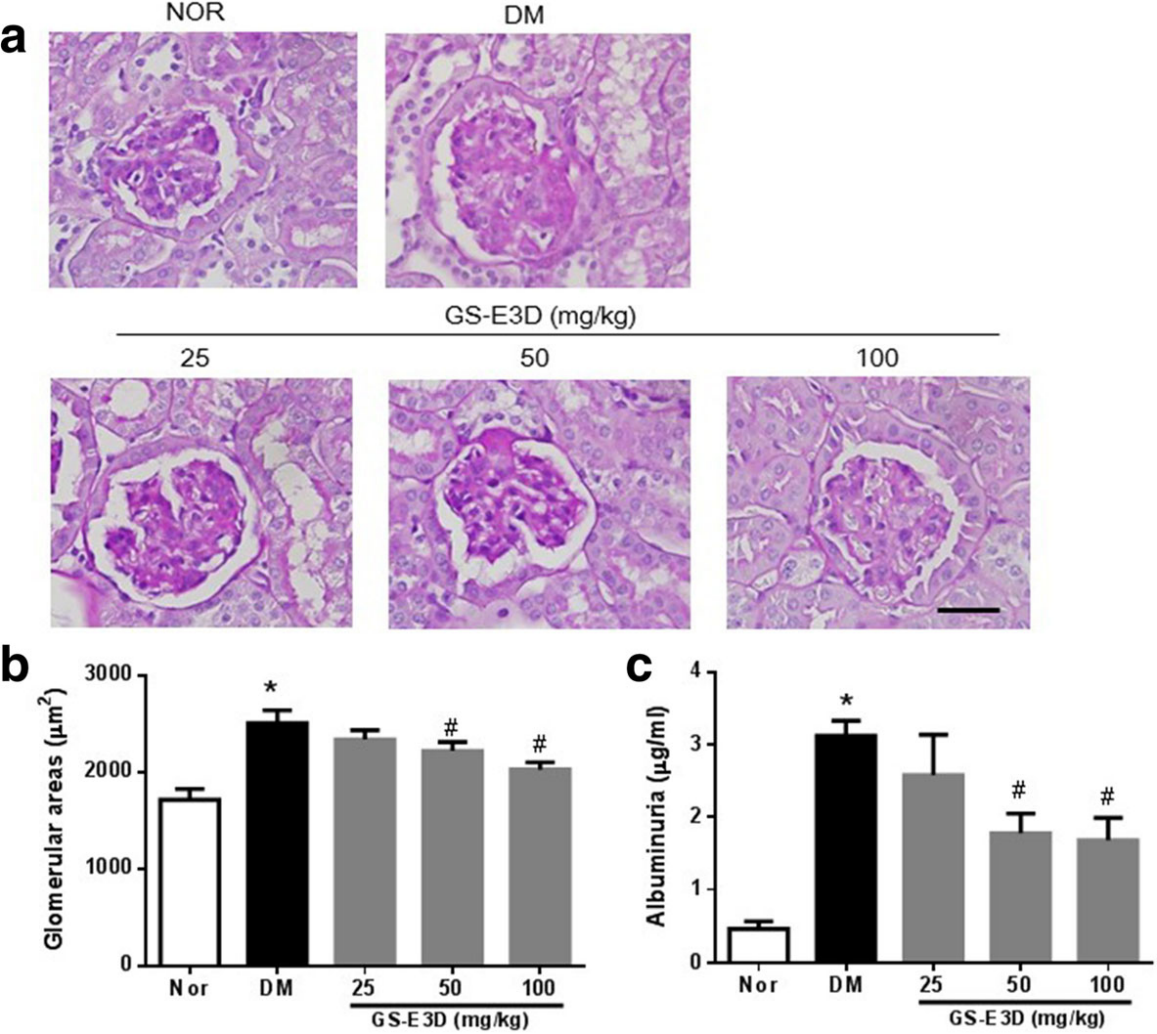
Renal histopathology and albuminuria. (**a**) Periodic acid-Schiff staining of glomeruli. Scale bar equals 50 μm. (**b**) Glomerular volume and (**c**) albuminuria in the indicated study groups. NOR, normal rat; DM, streptozotocin-induced diabetic rat; GS-E3D, DM treated with the indicated dose of GS-E3D. All data were expressed as mean ± the standard error of the mean (*n* = 8); *p < 0.05 vs. NOR group; #*p* < 0.05 vs. DM group

Eight weeks after the induction of diabetes, DM rats showed focal mesangial matrix expansion and tubulointerstitial damage (Fig. 1a). A significant enlargement of the glomeruli was observed in DM rats, as compared with the NOR group. In the GS-E3D-treated diabetic rats, glomerular size was dose-dependently smaller than that observed in the vehicle-treated diabetic rats (Fig. 1b). In addition, urinary albumin levels were significantly higher in the DM rats than in the NOR rats (*p* < 0.05), and these were dose-dependently decreased by the administration of GS-E3D (Fig. 1c).

### Oxidative status and renal tissue accumulation of AGEs

**Fig. 2:**
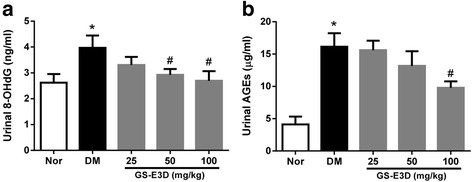
Urinary excretion of 8-hydroxy-2′-deoxyguanosine (8-OHdG) and advanced glycation end-products (AGEs). The urine levels of 8-OHdG (**a**) and AGEs (**b**) were determined by enzyme-linked immunosorbent assays. NOR, normal rat; DM, streptozotocin-induced diabetic rat; GS-E3D, DM treated with the indicated dose of GS-E3D. All data were expressed as mean ± the standard error of the mean (*n* = 8); *p < 0.05 vs. NOR group; #*p* < 0.05 vs. DM group

**Fig. 3:**
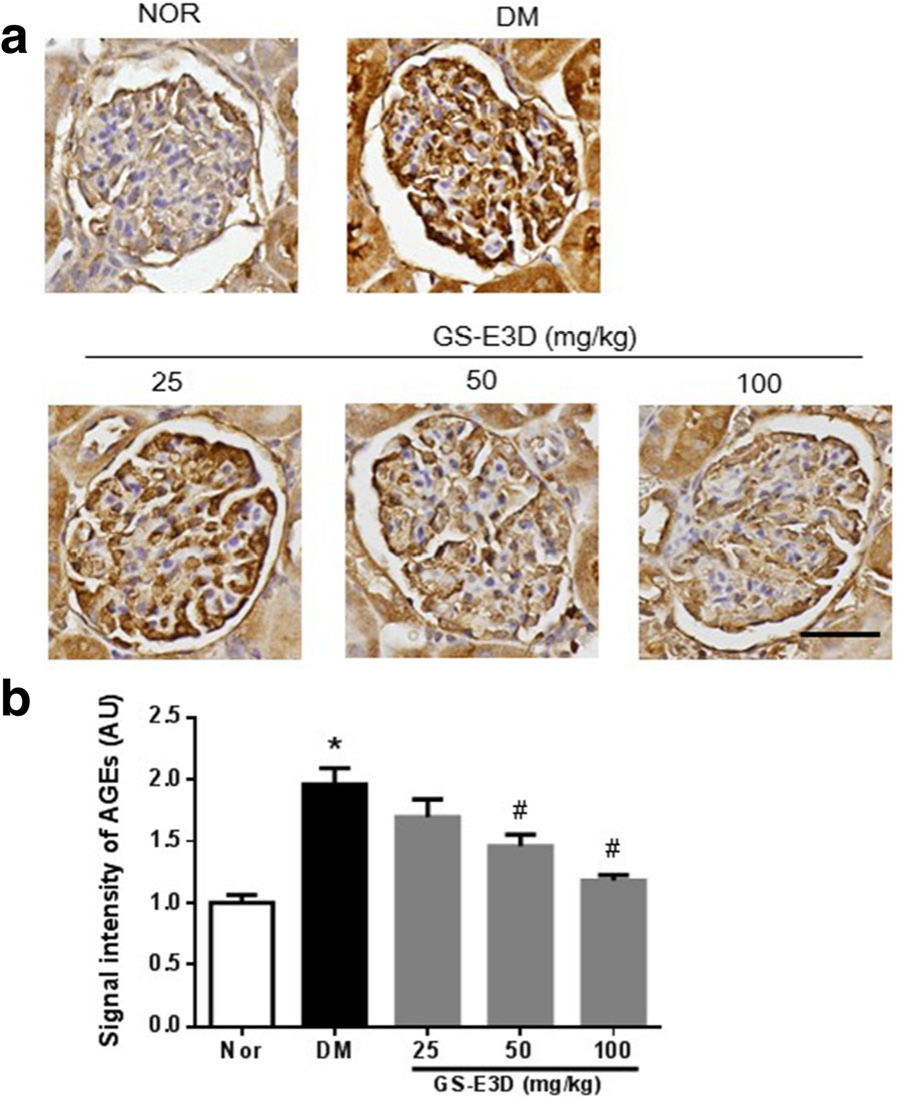
The effect of GS-E3D on renal accumulation of advanced glycation end-products (AGEs). (**a**) Immunohistochemical staining for AGEs. Scale bar equals 50 μm. (**b**) Quantification of the AGE signal intensity. NOR, normal rat; DM, streptozotocin-induced diabetic rat; GS-E3D, DM treated with the indicated dose of GS-E3D. All data were expressed as mean ± the standard error of the mean (*n* = 8); *p < 0.05 vs. NOR group; #*p* < 0.05 vs. DM group

The formation of AGEs is accelerated in diseases associated with increased oxidative stress [43]. AGEs exert harmful biological effects by activating RAGE [44]. Oxidative stress induced by interactions between AGEs and RAGE is involved in renal injury [13]. Thus, we performed urinary ELISAs for 8-OHdG in order to evaluate the damage caused by oxidative stress. The mean urinary 8-OHdG level in DM rats was significantly higher than that in NOR rats (Fig. 2a). This increase was dose-dependently inhibited in diabetic rats that were treated with GS-E3D. In addition, to determine renal accumulation of AGEs in diabetic rats, urinary AGE levels were determined. At the end of the study, these levels were significantly higher in DM rats than in NOR rats (Fig. 2b). Treatment with GS-E3D dose-dependently decreased urinary excretion of AGEs. Immunohistochemical staining for AGEs clearly showed accumulation in renal tissues of DM rats. Significantly higher levels of AGEs were present in these animals than in NOR rats (Figs. 3a and b). However, renal accumulation of AGEs was dose-dependently reduced in diabetic rats that were treated with GS-E3D.

### GS-E3D inhibits Mesangial cell proliferation

**Fig. 4:**
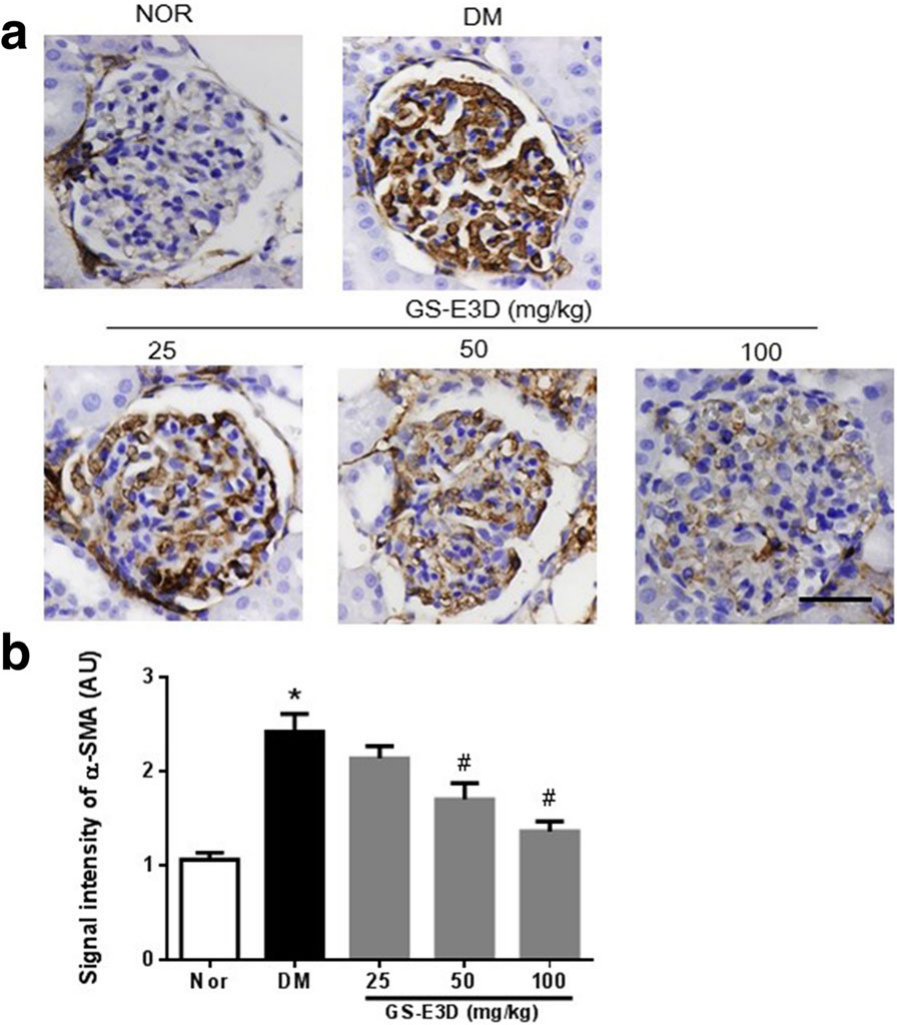
The effect of GS-E3D on α-smooth muscle actin (α-SMA) expression in glomeruli. (**a**) Immunohistochemical staining for α-SMA. Scale bar equals 50 μm. (**b**) Quantification of the α-SMA signal intensity. NOR, normal rat; DM, streptozotocin-induced diabetic rat; GS-E3D, DM treated with the indicated dose of GS-E3D. All data were expressed as mean ± the standard error of the mean (*n* = 8); *p < 0.05 vs. NOR group; #*p* < 0.05 vs. DM group

Diabetic nephropathy is characterized by glomerular hypertrophy, caused by the proliferation of mesangial cells [45]. α-SMA is a marker of mesangial cell proliferation [46]. To determine whether GS-E3D prevented mesangial proliferation, we performed immunostaining for α-SMA. Immunohistochemical staining of α-SMA in glomeruli demonstrated a significant increase in DM rats, as compared with the NOR group. This increase was dose-dependently inhibited in rats treated with GS-E3D (Figs. 4a and b).

## Discussion

GS-E3D is commercial pectin lyase-modified red ginseng extract with an enhanced level of the ginsenoside Rd. The results of this study showed that the administration of GS-E3D might ameliorate diabetic nephropathy in a rat STZ-induced model of diabetes. GS-E3D-treated diabetic rats showed significant improvements in markers of renal function, such as urinary albumin levels. In addition, GS-E3D reduced urinary 8-OHdG excretion and AGE accumulation in renal tissues, as well as inhibiting mesangial proliferation.

Hyperglycemia is present in STZ-induced diabetic rats, and diabetic nephropathy and mesangial expansion progress rapidly in this model [47]. Mesangial cells are smooth muscle-like contractile cells that are interspersed with the glomerular capillaries. Mesangial expansion by means of proliferation, hypertrophy, and matrix deposition is an early characteristic sign of diabetic nephropathy [48, 49]. Microalbuminuria is often the first clinical sign of renal dysfunction in patients with DM. Although albuminuria may partially result from defective reabsorption of proteins in the proximal tubule [50], it is associated with mesangial expansion in the majority of patients with DM [51]. Based on these findings, we hypothesized that GS-E3D might ameliorate mesangial expansion and thus reduce albuminuria. This protective effect of GS-E3D was partly attributed to its antioxidant and anti-AGE activities.

Hyperglycemia is an important causal factor that underlies the development of diabetic nephropathy. Recently, a type 1 diabetes mellitus (T1DM) animal model has often been used to study the mechanisms involved in diabetic complications behind the actions of anti-diabetic drugs independent of their glucose-lowering effects. For example, metformin and dipeptidyl peptidase IV (DPP4) inhibitors, such as vildagliptin and alogliptin, which is a well-known anti-diabetic drug for type 2 diabetes mellitus (T2DM), had no effect on blood glucose in the T1DM animal model [52–54]. Therefore, the purpose of this study was also to evaluate the effect of GS-E3D on diabetic nephropathy in a model without a blood glucose reduction. In our study, GS-E3D appeared protective effect on the diabetes-induced renal dysfunction independent of glycemic control. Additionally, Hong et al. showed that red ginseng lowered blood glucose levels in streptozotocin (STZ)-induced diabetic mice [30]. However, its oral dosage in mouse was 25 mg/mouse (1025 mg/kg; correspondence in a six-week old C57BL/6 mouse body weight of 20 g). In the present study, the highest dosage of GS-E3D (100 mg/kg) is about 10-fold less than that reported by Hong et al. Although, GS-E3D failed to decrease blood glucose in STZ-induced diabetic rat, GS-E3D has significant effects on the parameters of renal structure and function through inhibition of renal AGEs accumulation without the strong reduction of blood glucose.

Although various initiators of diabetic nephropathy have been proposed including glycation, the polyol pathway, and oxidative stress, one of the major consequences of hyperglycemia is the formation of AGEs. The formation of AGEs in renal tissue is closely correlated with the development of diabetic nephropathy [55, 56]. AGEs were previously reported to induce mesangial expansion and proteinuria [57]. The irreversible formation of AGEs damages the renal tissues and blood vessels [58]. Mesangial cells may be sensitive AGE targets because they express RAGE [59]. Consistent with this interpretation, our results identified mesangial expansion in the glomeruli that showed AGE accumulation in STZ-induced diabetic rats with albuminuria; treatment with GS-E3D ameliorated diabetes-associated renal dysfunction and AGE accumulation in this rat model.

ROS play an important role in the pathogenesis of diabetic nephropathy. Inhibition of ROS generation has been demonstrated to be effective in preventing the development and progression of diabetic nephropathy [60]. Moreover, Li et al. reported that antioxidants decreased high-glucose-induced ROS-related mouse mesangial cell dysfunction [61]. Enhanced generation of ROS was also induced by the interaction of AGEs with RAGE [62]. It was previously reported that red ginseng had antioxidant activity [63, 64]. Therefore, the reduction of urinary 8-OHdG levels by GS-E3D treatment may be due to its antioxidant and anti-AGE activities.

Several agents such as aminoguanidine and LR-90 have been proposed as AGE inhibitors, which attenuate mesangial expansion and proteinuria in diabetic animal models [15–17]. Our previous studies showed that some natural herbal products derived from *Polygonum cuspidatum* [65], Cassiae semen [66], or *Litsea japonica* [67] prevented diabetes-induced renal injury via inhibition of AGE accumulation in experimental animal models of diabetes. *P. ginseng* has been proven effective for anti-diabetes and anti-oxidation in many model systems [68]. In previous study, Korean red ginseng inhibited cyclosporine-induced renal injury [69]. Ginsenoside Rd attenuated renal dysfunction by preventing oxidative stress in cisplatin or cephaloridine-induced acute renal failure and ischemic-reperfused rats [70–72]. Although the efficacy of Korean red ginseng on diabetic nephropathy are poorly understood, Quan et al. reported that Korean red ginseng reduced the formation and secretion of AGEs from the kidneys of diabetic rats [32]. The ginsenoside Rd is one of the bioactive compounds present in red ginseng and this ameliorated astrocyte damage induced by methylglyoxal, an AGE precursor [73]. Yokozawa et al. showed that ginsenoside Rd also inhibited mesangial cell proliferation [74]. Unfortunately, we did not compare the effects of GS-E3D with those of an unmodified red ginseng extract. However, because GS-E3D had an enhanced level of Rd., as compared with an unmodified red ginseng extract [42], GS-E3D may have the more potent preventive effect on diabetes-induced renal dysfunction.

## Conclusion

Our data indicate that GS-E3D ameliorated diabetes-induced renal dysfunction in STZ-induced diabetic rats. We also demonstrated that GS-E3D protected these animals from ROS- and AGE-related renal injury. These effects could be attributed, at least in part, to reduced AGE deposition. However, the pharmacological mechanisms underlying the effects of GS-E3D need further investigation.

## Abbreviations

8-OHdG: 8-Hydroxy-2′-deoxyguanosine
AGEs: Advanced glycation end products
DM: Diabetes mellitus
ELISA: Enzyme-linked immunosorbent assay
NOR: Normal control
RAGE: Receptor for advanced glycation end products
ROS: Reactive oxygen species
STZ: Streptozotocin
α-SMA: α-smooth muscle actin
